# Trilayer Composite System Based on SiO_2_, Thiol-Ene, and PEDOT:PSS. Focus on Stability after Thermal Treatment and Solar Irradiance

**DOI:** 10.3390/polym13193439

**Published:** 2021-10-07

**Authors:** Algirdas Lazauskas, Dalius Jucius, Brigita Abakevičienė, Asta Guobienė, Mindaugas Andrulevičius

**Affiliations:** 1 Institute of Materials Science, Kaunas University of Technology, K. Baršausko 59, LT51423 Kaunas, Lithuania; dalius.jucius@ktu.lt (D.J.); brigita.abakeviciene@ktu.lt (B.A.); asta.guobiene@ktu.lt (A.G.); mindaugas.andrulevicius@ktu.lt (M.A.); 2 Department of Physics, Faculty of Mathematics and Natural Sciences, Kaunas University of Technology, Studentų Str. 50, LT51423 Kaunas, Lithuania

**Keywords:** SiO_2_, thiol-ene, PEDOT:PSS, trilayer, composite, thermal, solar irradiance, stability

## Abstract

The trilayer composite was fabricated by combining functional layers of fumed SiO_2_, thiol-ene, and poly(3,4-ethylenedioxythiophene) poly(styrenesulfonate) (PEDOT-PSS). Optical, scratch-healing, non-wetting, and electrical stability was investigated at different instances of time after thermal and solar irradiance treatment. The trilayer composite was found to be optically stable and highly transparent for visible light after thermal and irradiance treatment for 25 h. Both treatment processes had a minor effect on the shape-memory assisted scratch-healing performance of the trilayer composite. Thermal treatment and solar irradiance did not affect the superhydrophobic properties (contact angle 170 ± 1°) of the trilayer composite. The sheet resistance increased from 90 ± 3 Ω/square (initial) to 109 ± 3 Ω/square (thermal) and 149 ± 3 Ω/square (irradiance) after 25 h of treatment, which was considered as not significant change.

## 1. Introduction

Multilayer composites consisting of plane parallel layers represent an important class of functional and structural materials widely used today. Layered composites are of interest as each layer usually exhibits different functional properties, which add up to the whole system to meet the specific practical requirements for a certain application [1,2,3]. Functional multilayers are common in optics [4], electronics [5], and sensors [6]. Moreover, there has been a recent growing interest in advanced functional multilayers related to emerging applications in organic electronics [7,8]. Rapid development of organic electronics, triggered by the physical limitations of conventional silicon-based technologies, is expected to lead to a new generation of low-cost, light-weight, flexible, and transparent devices. According to a comprehensive research report by Market Research Future, “Organic Electronics Market, By Component (Active (Transistor, Sensor, Diode), Passive (Traces, Antenna)), By Material (Dielectric, Conductor, Luminescent), By Application (Display, Solar battery, Photovoltaic)—Forecast 2027” the market of organic electronics is expected to surpass USD 159.11 billion at a compound annual growth rate of 21% by 2027. Currently, this market is boosted by the increasing demand of high-performance semiconductors, flexible electronics, and organic light-emitting diode and photovoltaic applications. Herein, we designed a trilayer composite that could be integrated in all of these above-mentioned applications.

Poly(3,4-ethylenedioxythiophene) poly(styrene sulfonate) (PEDOT:PSS) is recognized as the most popular and commercially most successful conductive polymer in organic electronics as well as a promising material for excellent performance Si/PEDOT:PSS heterojunction solar devices [9,10,11]. This conductive polymer exhibits a number of exceptional properties, e.g., thermal stability, physical and chemical stability in air, good film forming ability, high flexibility, solubility in water, and high optical transparency. The polymer is usually coated on polydimethylsiloxane, polyimide, or other conventional substrate protecting from the environment [12,13]. Such substrates are not resistant to accidental scratches that degrade their optical performance. Therefore, self-healing layers, such as thiol-ene, are very promising to solve this problem [14]. Thiol-ene falls in a group of intrinsic self-healing materials capable of healing multiple times and does not contain vascular networks or microcapsules which negatively affect optical transmittance of the material [15]. UV polymerizable thiol-ene layers exhibit low volume shrinkage, flexibility, high optical transparency, toughness, homogeneity of the polymer network, and negligible oxygen inhibition [16,17,18]. It was shown previously in [19] that thiol-ene films made of pentaerythritol tetrakis(3-mercaptopropionate) (PETMP) and 1,3,5-triallyl-1,3,5-triazine-2,4,6(1H,3H,5H)-trione (TTT) photopolymerizable composition exhibit shape-memory properties and are capable of recovering their original shape after a temporary deformation when external stimulus is applied, which is very useful for a specific self-healing mechanism based on the bridging of the fractured surfaces into close proximity [20]. Further enhancement of optical properties of the layered composites can be achieved by adding an extra layer on the top with suitable interfacial nanostructures [21]. High optical transmittance, as well as superohydrophobicity and self-cleaning properties, are inherent to thin silica-based layers composed of SiO_2_ nanoparticles [22,23]. Such layers can be efficiently deposited from SiO_2_ nanoparticle dispersions [24] by various thin layer fabrication methods, e.g., dip-coating, spray-coating, spin-coating, and Langmuir–Blodgett deposition. In response to the growing market demand for similar products, we have designed and tested a novel trilayer composite which could be integrated in modern photovoltaic modules, displays, and other devices of organic electronics. Thiol-ene thermoset polymer was selected as a base layer, PEDOT:PSS conductive polymer as a bottom layer, and finally SiO_2_-based nanostructured superhydrophobic material was deposited as a top layer in a trilayer composite system.

In this communication, we report on the optical, scratch-healing, non-wetting, and electrical stability of trilayer composite (SiO_2_/thiol-ene/PEDOT:PSS), which was subjected to thermal and solar irradiance treatment. Our study has demonstrated that the fabricated trilayer composite is stable enough and assembles the above-mentioned functional properties of each individual layer. The superhydrophobicity, optical transparency, electrical conductivity, and self-healing properties of the trilayer composite make it an exceptional material for optoelectronics. To the best of our knowledge, this is the first attempt to fabricate a trilayer composite with particular multifunctional properties and systematically evaluate its stability after thermal and solar irradiance treatment. There are no similar works reported in the literature accomplishing similar trilayer composite system. Specific applications where this composite can be directly used include, but are not limited to, all-solution processed transparent organic light emitting diodes [25], and polymer solar cells [26]. The PEDOT:PSS layer would act as an electrode in a layered device structure, while the SiO_2_ and thiol-ene layers would protect from the accumulation of dust particles and scratches that decrease the device efficiency.

## 2. Materials and Methods

### 2.1. Materials

1,3,5-triallyl-1,3,5-triazine-2,4,6(1H,3H,5H)-trione (TTT, trifunctional allyl component, ≥ 97.5%), pentaerythritol tetrakis(3-mercaptopropionate) (PETMP, tetrafunctional thiol component, > 95%), 2,2-dimethoxy-2-phenylacetophenone (DMPA, photoinitiator, ≥ 98.5%), potassium hydroxide (KOH, ≥ 85.0%), 3,4-ethylenedioxythiophene (EDOT, ≥ 96.5%), poly(sodium 4-styrenesulfonate) (PSS, > 95%), sodium persulfate (Na_2_S_2_O_8_, ≥ 99%), ferrous sulfate heptahydrate (FeSO_4_·7H_2_O, ≥ 99%), hexamethyldisilazane (HMDS, ≥ 99%), glycerol (GLY, ≥ 99.5%), ethylene glycol (EG, ≥ 99.8%), polyethylene glycol (PEG, ~50% in H_2_O), and fumed SiO_2_ (CAS 112945-52-5, surface area 175–225 m^2^/g, 99.8%) were purchased from Sigma-Aldrich (St. Louis, MO, USA). Ethanol (96%) was purchased from MV Group Production (Kaunas, Lithuania). All reagents were used without further purification. Ultrapure water with a resistivity higher than 18.2 MΩ/cm at 25 °C was used in all experiments, and was obtained from a Direct-Q^®^ 3 UV water purification system (Merck KGaA, Darmstadt, Germany).

### 2.2. Fabrication of the Trilayer Composite

The photopolymerizable thiol-ene composition was prepared as a mixture of PETMP and TTT with a 1:1 stoichiometric ratio of thiol to ene functional groups, containing 1 wt% of DMPA. Details of the preparation procedure have been previously reported [20]. The clear, colorless, viscous mixture of PETMP and TTT was applied on polytetrafluoroethylene (PTFE) plate as a 100 ± 1 μm-thick layer via the Meyer rod coating method. All samples were UV cured simultaneously at intensities of 1.64 mW/cm^2^ (254 nm wavelength) and 0.8 mW/cm^2^ (365 nm wavelength) for 5 min. Free-standing PETMP-TTT films were obtained by gently peeling the film from the PTFE plate. The water dispersions of PEDOT:PSS colloids were synthesized by oxidative polymerization of EDOT in the presence of the PSS. Details of the synthesis procedure have been previously reported [27]. PEDOT:PSS dispersions containing DMSO (50 μL/mL) were spin-coated on the one side of the free-standing PETMP-TTT at 2000 rpm for 30 s (layer thickness ~50 ± 10 nm). Subsequently, it was dried at room temperature for 24 h and cured on a hot plate at 125 °C for 2 min. Prior to the spin-coating procedure, the PETMP-TTT side to be coated with PEDOT:PSS was exposed to O_2_ radio frequency (RF) plasma in the camera of the device Plasma-600-T (JSC Kvartz, Kaliningrad, Russia) at 133 Pa pressure (RF = 13.56 MHz, P = 0.3 W/cm^2^, t = 30 s) in order to improve the wetting and adhesive characteristics of the surface. Details of the silylation derivatization reaction of fumed SiO_2_ with HDMS, as well as the preparation of the nanoparticle dispersion, have previously been reported [24]. The later was spin-coated on another side of PETMP-TTT at 3000 rpm for 30 s (layer thickness ~30 ± 10 nm). Subsequently, it was dried on a hot plate at 80 °C for 5 min. The resultant trilayer composite was denoted as SiO_2_/PETMP-TTT/PEDOT:PSS.

### 2.3. Characterization

Atomic force microscopy (AFM) experiments were carried out at room temperature using a NanoWizardIII atomic force microscope (JPK Instruments, Bruker Nano GmbH, Berlin, Germany), while the data were analyzed using a SurfaceXplorer and JPKSPM Data Processing software (Version spm-4.3.13, JPK Instruments, Bruker Nano GmbH, Berlin, Germany). AFM images were collected using an ACTA (Applied NanoStructures, Inc., Mountain View, CA, USA) probe (tip shape: pyramidal, radius of curvature (ROC): <10.0 nm, and cone angle: 20°; silicon cantilever shape: pyramidal, reflex side coating: Al with thickness of 50 ± 5 nm, calibrated spring constant: 54.2 N/m, and set point: 195.48 nN) operating in contact quantitative imaging mode. The values of the tip radius and tip materials were taken from datasheets provided by the manufacturer of the probes.

An optical spectrometer Avantes that is composed of a deuterium halogen light source (AvaLight DHc, Avantes, Apeldoorn, The Netherlands) and a spectrometer (Avaspec-2048, Avantes, Apeldoorn, The Netherlands) was used to record UV-visible light transmission spectra.

Scratch testing of the trilayer composite was performed with a custom-made PC controlled scratch testing apparatus. During the scratch test, the samples were scratched (scratch length: 10 mm and speed: 0.2 mm/s) with a spheroconical stylus (cone angle: 90° and indenter radius: 45 μm) applying the constant loading of 1.5 N. The scratches were performed in air atmosphere (temperature: 23 °C and humidity: 40%). The B-600MET series upright metallurgical microscope (OPTIKA Srl, Ponteranica, Italy) with a c-mount 2560 × 1920 resolution (5.0 Mpixel) camera (Optikam Pro 5LT) was used for the inspection of the scratch track.

Four-point probe testing system (Ossila Ltd., Sheffield, UK) was used for the sheet resistance measurements. The probe spacing was 1.27 mm, the target current 10 mA, the maximum voltage 5 V, and the voltage increment 0.010 V. Measurements were performed at room temperature and a relative humidity of ~40%.

Contact angle (CA, θ) measurements were performed at room temperature using the sessile drop method. A droplet of deionized water (5 μL) was deposited onto the investigated surface. Optical images of the droplet were recorded with a PC-connected digital camera after 10 s of dropping and CA measurements were carried out using an active contour method based on B-spline snakes [28]. CA hysteresis was measured as the difference between the advancing and receding contact angle of a sliding droplet. Surface free energy (γ) was estimated by measuring the CA of three different solvents (i.e., GLY, EG, and PEG). An average value of CA obtained from the Young–Laplace fitting approach was used for calculation of the γ and its polar γ_p_ and disperse γ_d_ components employing Owen–Wendt–Rabel–Kaelble method.

### 2.4. Treatment

**Figure 1:**
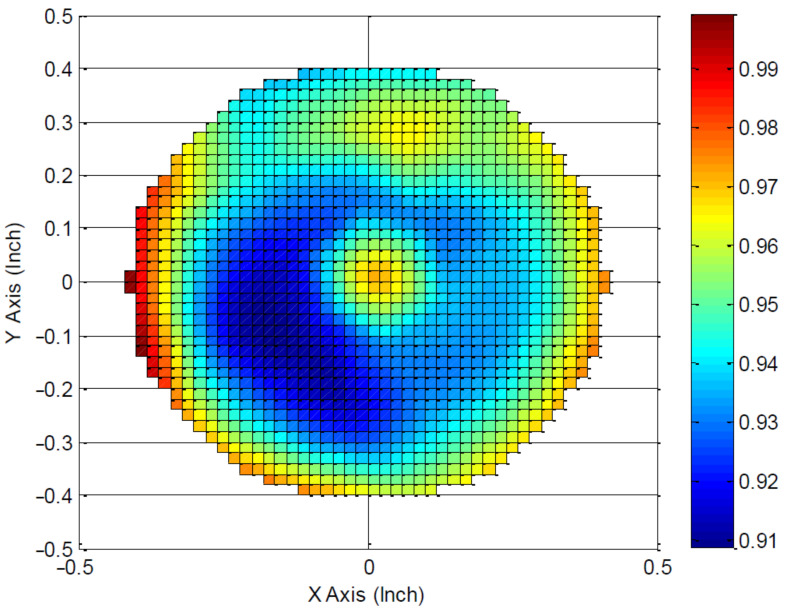
Irradiance uniformity map of solar spectrum simulator SF150-B with air mass filter AM1.5G.

The solar spectrum simulator SF150-B (Scientech Inc., ON, Canada) was used as a broadband light source for irradiance of the samples. Air mass filter AM1.5G (Scientech Inc., Ontario, Canada) with spectral range of 300–2000 nm was used in the experiments. The irradiance power (with filter) was 1 Sun equal to 100 mW/cm^2^. The irradiance uniformity map interpreted from measured data is shown in Figure 1. Measured non uniformity (NU) was 4.34 %, which corresponds to Class B non-uniformity. Thermal treatment was performed on a POLOS Hotplate 200S (APT GmbH, Bienenbüttel, Germany).

## 3. Results and Discussion

It was shown by our group that the PETMP-TTT/PEDOT:PSS composite, consisting of plane parallel layers PETMP-TTT and PEDOT:PSS, is highly transparent for visible light [24]. The choice of incorporation of amorphous SiO_2_ as a superhydrophobic top layer in the trilayer composite system was related to its excellent thermal, insulation, chemical stability, and optical properties [29,30].

**Figure 2:**
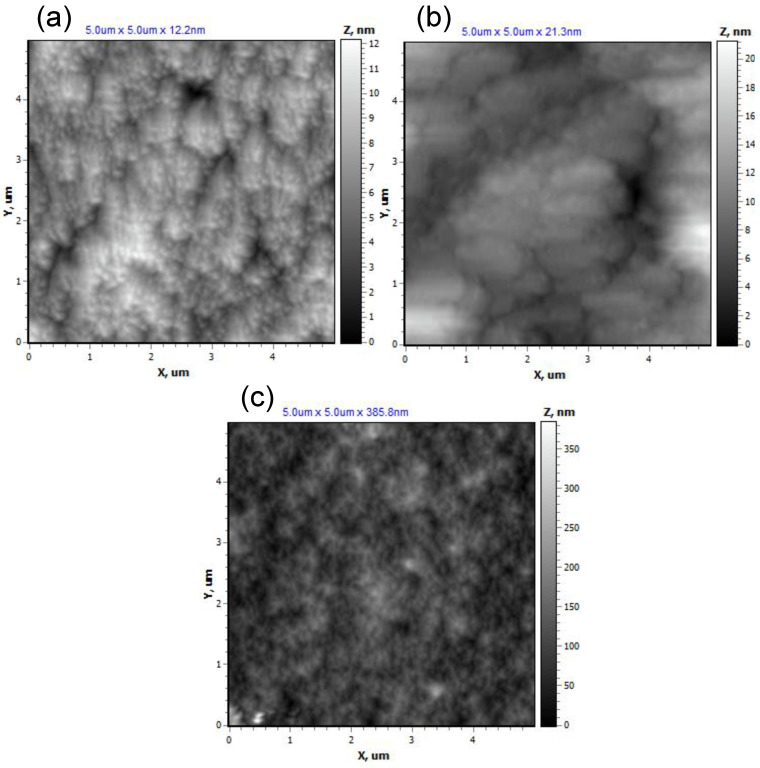
AFM surface topography of (**a**) PETMP-TTT, (**b**) PEDOT:PSS, and (**c**) SiO_2_ functional layers.

AFM analysis of the PETMP-TTT, PEDOT:PSS, and SiO_2_ functional layers (Figure 2a–c) was conducted over 5.0 × 5.0 μm^2^ area for quantitative morphological evaluation. The surface structures of PETMP-TTT have a mean height (*Z*_mean_) of 6.26 nm with a root mean square roughness (*R*_q_) of 1.52 nm. The surface topography is characterized by larger segmental elements (width in the range of 200–400 nm), covered with fine-grained structures (20–30 nm). The surface is symmetric: surface peaks dominate over the valleys with a skeweness (*R*_sk_) value of 0.07 and exhibit a leptokurtoic distribution of the morphological features with a kurtosis (*R*_ku_) value of 3.36. The *Z*_mean_ and *R*_q_ values for PEDOT:PSS deposited on PETMP-TTT were found to be 8.77 nm and 2.75 nm, respectively. The surface topography of PEDOT:PSS partially repeats that of PETMP-TTT with slight increase in roughness. The topography of PEDOT:PSS follows similar distribution of surface morphological features with *R*_sk_ and *R*_ku_ values of 0.92 and 4.96, respectively. It is important to note that, for the manufacturing of optical surfaces, an adequately low surface roughness is crucial [31], which is the case of the PETMP-TTT and PEDOT:PSS layers. A significant increase in surface roughness was observed for the SiO_2_ functional layer deposited on the PETMP-TTT with *Z*_mean_ and *R*_q_ values of 96 nm and 32.92 nm. The surface topography of SiO_2_ has no correlation with that of PETMP-TTT. The surface is rough and asymmetric. Herein, surface peaks also dominate over the valleys with *R*_sk_ value of 0.73; the *R*_ku_ was found to be 5.03. It was previously reported, that for the combination of high optical transmittance and superhydrophobicity to work, surface roughness should be adjusted to submicrometer scale [24], in agreement with AFM results.

**Figure 3:**
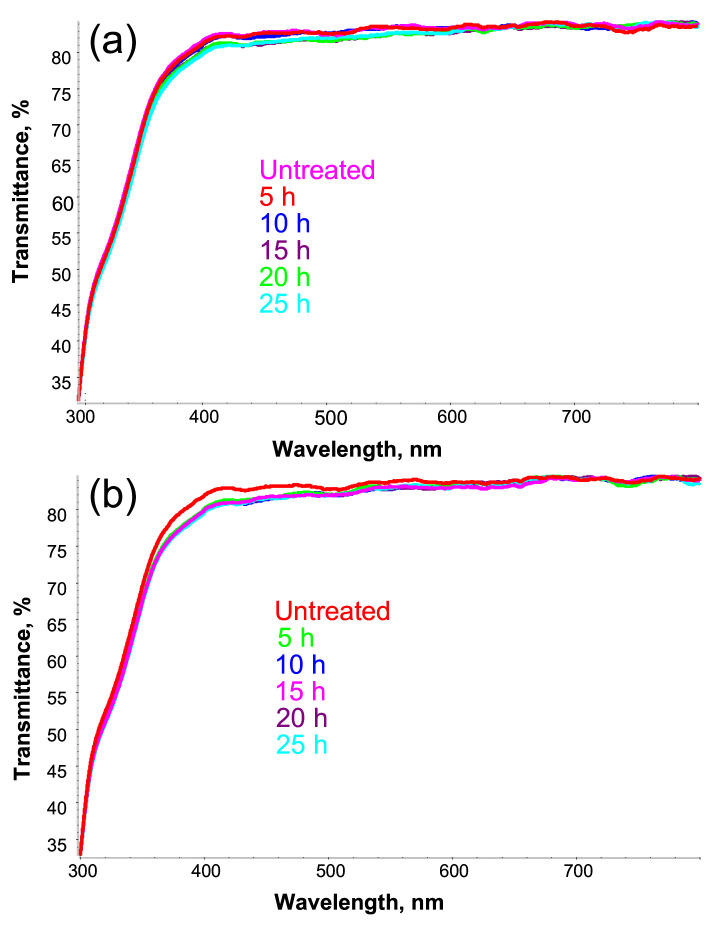
UV-visible transmittance of the trilayer composite after (**a**) thermal (120 °C) and (**b**) solar irradiance treatment for different time intervals.

The optical stability of the fabricated SiO_2_/PETMP-TTT/PEDOT:PSS trilayer composite was tested employing thermal and solar irradiance treatment. Figure 3a,b show UV-visible transmittance spectra of the trilayer composite before and after thermal (120 °C) and irradiance treatment for different time intervals, respectively. Only a slight decrease in the transmittance of wavelengths in the range of 350–600 nm was observed after thermal and irradiance treatment of SiO_2_/PETMP-TTT/PEDOT:PSS for 25 h, thus the resultant trilayer composite can be considered optically stable and highly transparent for visible light.

**Figure 4:**
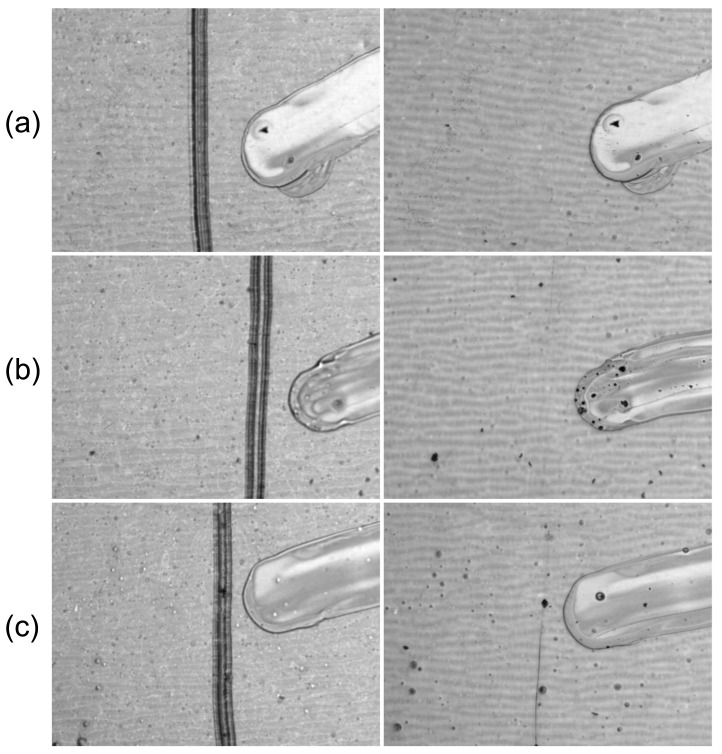
Shape-memory assisted scratch-healing stability of the trilayer composite: optical microscope digital photographs at 75× magnification of (**a**) untreated sample as well as after (**b**) thermal and (**c**) irradiance treatment for 25 h (left) before and (right) after healing process of the scratch produced with constant loading of 1.5 N.

In another experiment, the trilayer composites were subjected to thermal (120 °C) and solar irradiance treatment for 25 h. Subsequently, the SiO_2_/PETMP-TTT/PEDOT:PSS samples were cooled down below 0 °C and left to stand at a room temperature for 5 min. This procedure was performed to ensure improved form fixity of the PETMP-TTT layer via immobilization of the polymer chains. Finally, scratch testing was performed to evaluate the scratch-healing ability of the composite films. Figure 4 shows optical microscope digital photographs of a scratch produced with constant loading of 1.5 N on the untreated trilayer composite, as well as after thermal (120 °C) and irradiance treatment for 25 h, before and after the healing process (5 min at 70 °C). As it is evident from Figure 4, thermal and irradiance treatment had a minor effect on shape-memory assisted scratch-healing performance of the trilayer composite. Only minor scratches were observable after the healing process of the thermally treated and irradiated trilayers (Figure 4b,c), indicating excellent shape-memory assisted scratch-healing stability of the fabricated trilayer composite. This finding is consistent with the results of previous studies, showing excellent scratch-healing, shape fixity, and recovery properties of this particular composition of PETMP-TTT [19,20,32].

**Figure 5:**
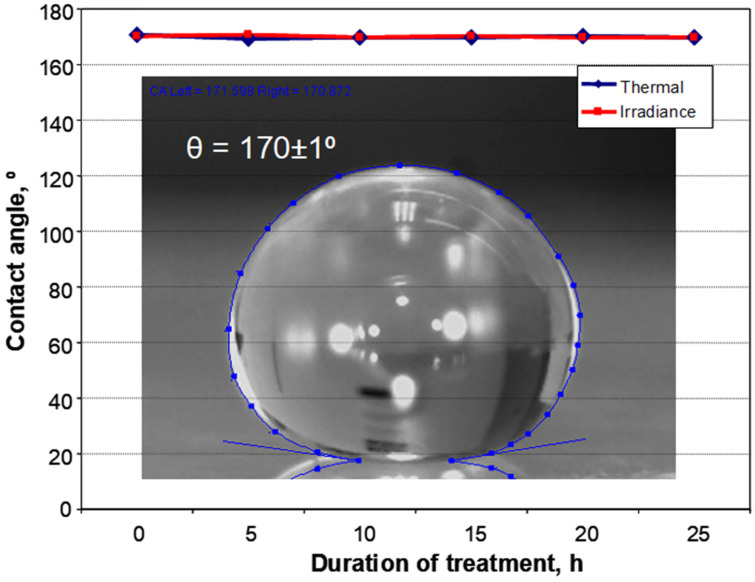
Stability of superhydrophobicity of the trilayer composite: contact angle after thermal and irradiance treatment for different time intervals. The inset shows the characteristic digital photograph of the magnified 5 μL water droplet on the surface of the trilayer composite.

Figure 5 shows the changes in CA of the 5 μL water droplet on the surface of the trilayer composite after thermal (120 °C) and solar irradiance treatment for different time intervals. The SiO_2_ layer retained its superhydrophobic properties after 25 h of thermal and irradiance treatment with stable CA of 170 ± 1°. The determined CA hysteresis was ≤2° in all cases. Stability of the superhydrophobic state can be attributed to the combination of suitable surface chemistry and the specific surface architecture of the nanostructured SiO_2_ layer that ensures the Cassie–Baxter wetting regime [24,33,34,35]. The surface free energy (γ) and its polar (γ_p_) and disperse (γ_d_) components for SiO_2_ layer were found to be 4.3 ± 0.172, 1.2 ± 0.05, and 3.0 ± 0.12 mN/m, respectively.

**Figure 6:**
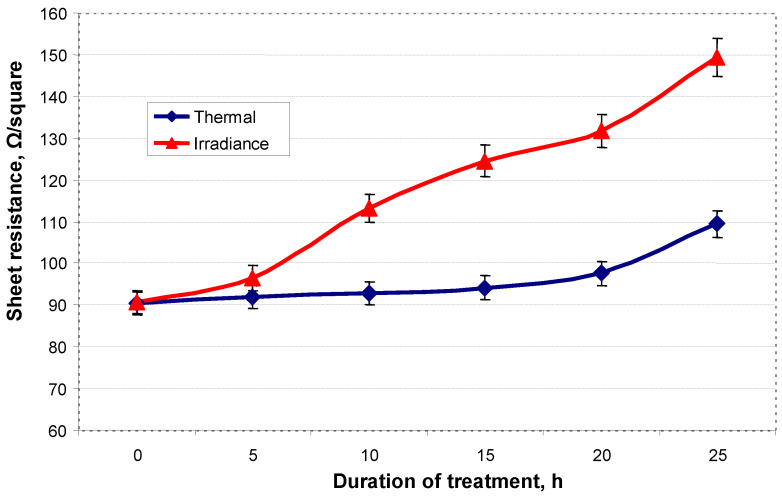
Electrical stability of the trilayer composite: sheet resistance after thermal and irradiance treatment for different time intervals.

Figure 6 shows the changes in the sheet resistance of the trilayer composite after thermal (120 °C) and solar irradiance treatment for different time intervals. The untreated trilayer composite exhibited sheet resistance as low as 90 ± 3 Ω/square. Thermal treatment from 0 to 20 h at 120 °C resulted in a slight increase in sheet resistance up to 97 ± 3 Ω/square. Sheet resistance of 109 ± 3 Ω/square was measured after 25 h of thermal treatment. Vitoratos et al. reported on the thermal degradation mechanisms of PEDOT:PSS [36]. It was demonstrated that PEDOT:PSS undergoes conformational changes with time during thermal treatment. These changes are responsible for the deterioration of the electrical properties of PEDOT:PSS. The irradiance treatment of the trilayer composite had a more pronounced impact on the electrical stability of the trilayer composite. The gradual increase in sheet resistance from 113 ± 3 Ω/square to 149 ± 3 Ω/square was determined for the irradiance treatment time in the range of 10–25 h. In another study by Elschner et al., the stability of PEDOT:PSS was investigated as a function of photon energy and exposure time [37]. It was reported that, upon irradiance treatment, the sheet resistance of PEDOT:PSS increases due to interruption of the free charge carried percolation arising from oxidative processes within the PEDOT-chains. The PEDOT:PSS layer in the trilayer composite system is considered as the bottom layer, thus it is protected by PETMP-TTT and SiO_2_ layers. In optoelectronic devices, this protective encapsulation of PEDOT:PSS would eliminate the oxygen and humidity contact in the ambient, which is responsible for electrical conductivity degradation [10]. Finally, the change in sheet resistance in both cases is not significant and may satisfy the operational prerequisites for numerous optoelectronic devices.

## 4. Conclusions

The trilayer composite was fabricated by combining fumed SiO_2_, PETMP-TTT, and PEDOT:PSS. Quantitative morphological evaluation was performed using AFM, giving information on surface topography and roughness of each individual layer. The fabricated trilayer composite was subjected to thermal and solar irradiance treatment, and the optical, scratch-healing, non-wetting, and electrical stability of the trilayer composite was investigated. The trilayer composite was found to be optically stable and highly transparent for visible light: only a slight decrease in the transmittance of wavelengths in the range of 350–600 nm was observed after thermal and irradiance treatment for 25 h. Thermal and irradiance treatment had a minor effect on the shape-memory assisted scratch-healing performance of the trilayer composite. The trilayer composite exhibited superhydrophobic properties with CA of 170 ± 1° and CA hysteresis of ≤2°, retained stability of superhydrophobic properties after both treatment processes. The changes in sheet resistance were considered to be not significant, as the sheet resistance increased from the initial 90 ± 3 Ω/square to 109 ± 3 Ω/square (thermal) and 149 ± 3 Ω/square (irradiance) after 25 h of treatment.

## Data Availability

The data will be available from the authors upon request.
